# Can science fiction engagement predict identification with all humanity? Testing a moderated mediation model

**DOI:** 10.3389/fpsyg.2022.943069

**Published:** 2022-08-18

**Authors:** Fuzhong Wu, Mingjie Zhou, Zheng Zhang

**Affiliations:** ^1^School of Journalism and Communication, Tsinghua University, Beijing, China; ^2^Institute of Psychology, Chinese Academy of Sciences, Beijing, China; ^3^Department of Psychology, University of Chinese Academy of Sciences, Beijing, China

**Keywords:** identification with all humanity, science fiction, narrative persuasion, construal level, actively open-minded thinking

## Abstract

Identification with all humanity (IWAH) is viewed as a critical construct that facilitates global solidarity. However, its origins have rarely been explored in previous literature, and no study has yet investigated the role of pop-culture in cultivating IWAH. To address this gap, this study initially focuses on science fiction (sci-fi), a specific pop-culture genre with worldwide audiences, and examines its effect on IWAH. It hypothesized a direct association between sci-fi engagement and IWAH from the narrative persuasion approach, and an indirect association via abstract construal based on the cognitive-literary approach. Moreover, the moderating role of actively open-minded thinking (AOT) in the direct and indirect association was also assessed. Results were obtained through a cross-sectional survey conducted in China (*n* = 570) and showed that sci-fi engagement was positively associated with IWAH; this association was also partially mediated by abstract construal. Interestingly, and inconsistent with our hypotheses, AOT positively moderated the indirect effect but negatively moderated the direct effect. Theoretical and practical implications for cultivating IWAH from the media and pop-cultural perspective were discussed.

## Introduction

Unsolved global crises such as COVID-19, climate change, depleting natural resources, terrorism and regional conflicts have posed severe threats and challenges to the collective future of human society. Apart from scientific, technological, and political solutions, global solidarity which underpins joint efforts and collective actions to combat these problems have become more urgent and necessary. To this end, scholars have suggested that identification with all humanity (IWAH)—a construct defined as psychological bonds with people all over the world and a concern for all members of the human community—may drive people to think and act globally. Empirically, IWAH was found to be positively associated with concern for humanitarian needs, globalism, and intergroup forgiveness (McFarland et al., 2012; Hamer et al., 2018), and negatively associated with intergroup dehumanization, and ethnocentrism (McFarland et al., 2019). Moreover, people with higher IWAH are more likely to engage in globally responsible behaviors, such as supporting international charities, fighting against global inequality, and health and prosocial behaviors during the COVID-19 pandemic (Reese et al., 2012; McFarland, 2017; Barragan et al., 2021; Deng, 2021; Marchlewska et al., 2022).

However, cultivating IWAH can be challenging. It requires individuals to lower self-centeredness and transcend subordinated social identities (e.g., national identities, ethnical identities) to connect with unfamiliar people who are far away. Compared to the ample studies on the social consequences of IWAH, knowledge about its origins is relatively lacking. To address this, recent studies have revealed that individual differences such as openness to experiences, empathy, and cognitive factors such as cognitive complexity may be behind IWAH (McFarland et al., 2012; Hamer et al., 2018, 2019). Inspiringly, some studies suggested that cultural activities such as intercultural contact, multi-cultural experiences, and mind-body practices are linked to high IWAH (Sparkman and Eidelman, 2018; Loy and Reese, 2019; Sparkman and Hamer, 2020). However, no study has examined the relationship between pop-culture engagement and IWAH, largely neglecting the affordance of media and cultural resources in connecting people all over the world.

Of all genres of pop-culture, this study focuses on science fiction (sci-fi) and examines whether, why, and when sci-fi engagement fosters IWAH. Sci-fi is a genre of speculative fiction that portrays imaginative and futuristic issues based on postulated scientific discovery and technological advancement. In recent years, sci-fi has become one of the dominating genres in films, literature, TV series, video games, and other media, and is consumed, discussed, and disseminated by people worldwide (Wells, 2013). According to the Internet Movie Database (IMD) (2022), as of March 2022, seven of the top ten films that have received the highest lifetime grosses are sci-fi films (e.g., *Avatar* [2009], *Avengers*: *Endgame* [2019]). In a survey across different countries (*n* = 902), 92.1% of the participants indicated they liked sci-fi/fantasy films and TV shows, and 46.2% agreed that sci-fi/fantasy “is the best thing ever” (Menadue and Jacups, 2018). In China, where the current study was carried out, the total value of sci-fi industries, which include subindustries of sci-fi film, publication, video game, and tourism, reached 55.109 billion RMB in 2020, according to the Chinese Research Institution for Science Popularization (2021). Given the worldwide popularity of sci-fi, it is interesting and essential to question whether it can bridge people across the world. If the association of sci-fi engagement and IWAH can be established, we may expect a media and cultural strategy for cultivating people’s IWAH across different countries.

Therefore, we proposed two disparate approaches that may explain the association between sci-fi engagement and IWAH. From the narrative persuasion approach, we hypothesized that sci-fi, a genre which portrays humanity as a whole and cares for their living conditions, may have a positive and direct persuasive effect on individuals’ IWAH. From the cognitive-literary approach, we hypothesized that sci-fi, a genre distancing people from their experienced reality, would foster abstract construal and consequentially positively predict IWAH. Furthermore, given the fictional and imaginative nature of sci-fi, which comprises knowledge and anticipations that may contradict with people’s existing belief, we hypothesized that actively open-minded thinking (AOT) may serve as a moderator both in the direct and indirect association. A cross-sectional survey (*n* = 570) was conducted in China and the hypotheses were tested through a moderated mediation model. The results provide preliminary evidence on the association between sci-fi engagement and IWAH, which may not only enrich our understanding of the cognitive and social impacts of sci-fi, but also suggest a new direction for cultivating IWAH.

## Theoretical background and hypothesis development

### Identification with all humanity

According to social-categorization theory (Turner et al., 1987), people can categorize themselves into different social groups based on the levels of abstraction, resulting in various self-definitions. For instance, at the lowest level, people may see themselves as unique individuals; at the middle-ranged levels, people may define themselves as members of a family, city, or nation; and at the highest level, people may feel that they belong to the human community (Reicher et al., 2010). When self-defined as a part of all humanity, people would expand their concern from their national, regional, and ethnical groups to the human race in general, even the unborn future generations (McFarland and Brown, 2008). In this regard, identifying with humanity is viewed as one of the most essential parts of achieving the highest level of human needs, namely, self-actualization, and the most mature and far-reaching form of social interest (Maslow, 1954; McFarland et al., 2012). To capture this highest order of human need and social interest, McFarland and Brown (2008) proposed the concept of identification with all humanity (IWAH, or global human identification) and defined it as “a positive caring, a genuine concern and love for all other members of the human family, and a regarding of all other human beings as part of one’s ingroup” (p. 39). Previous literature has examined IWAH both as a situationally activated factor and a dispositional characteristic. The former approach suggests that human identity can be elicited through experimental manipulation, such as portrayals of people with diverse national and ethnic backgrounds getting together (Greenaway et al., 2011; Reese et al., 2015; Sparkman et al., 2022). Moreover, when the superordinate identity of humanity is made salient, as supported by the Common Ingroup Identity Model (CIIM), people tend to disregard their subordinate social identities and recategorize themselves into the more inclusive group, which in turn, may alter people’s attitudes and behavioral intention toward the former out-group members (Gaertner et al., 1993; Greenaway et al., 2011; Gaertner and Dovidio, 2014). Studies from the latter approach, however, view IWAH as an individual difference and have developed a psychometric scale to measure it (McFarland et al., 2012; Hamer et al., 2019). Preliminary studies suggested that all items of the IWAH scale loaded on one dimension (McFarland et al., 2012), and a two-dimension model was also found for other samples in several countries (e.g., McFarland and Hornsby, 2015; Reysen and Hackett, 2016). The two dimensions were labeled as: (1) global self-definition (or bond with all humanity), reflecting a sense of membership in the human community, and (2) global self-investment (or concern for all humanity), referring to one’s commitment and tendency to devote to the group of all humanity, respectively (Reese et al., 2015; Hamer et al., 2021). Compared to people with low trait IWAH, those with high trait IWAH have greater concern for humanitarian needs, international human rights, and global issues (e.g., AIDS, global inequality), and exhibit more prosocial international attitudes and behaviors (McFarland, 2015, 2017; McFarland and Hornsby, 2015; for a review, see McFarland et al., 2019).

In essence, developing IWAH involves the process of transcending one’s specific social circumstances and connecting to the distant others, as well as the human community, both emotionally and cognitively. Although it is impossible for people to know or get in touch with everyone across the world, and the notion of humankind might be too abstract to comprehend, people may rely on their imagination to make sense of the larger social group beyond their direct experiences. As noted by Anderson (1983), all large communities are imagined in their nature. Moreover, in contemporary societies, media and cultural experiences, including pop-cultural experiences, play a vital role in constructing this collective imagination and shaping their way of construing the world, and profoundly impacts on the process of identity formation (Siriyuvasak and Hyunjoon, 2007; Plante et al., 2014; Cui et al., 2016). In light of this, we switch to the media and pop-cultural resources, and particularly to whether sci-fi genre, which has a worldwide audience, can help to cultivate IWAH.

### Sci-fi engagement and identification with all humanity

While sci-fi writers, audiences, and theorists may understand sci-fi from different perspectives, some consensus has been reached. The most essential definition of sci-fi was proposed by Suvin (1972), who regarded it as a genre of “cognitive estrangement.” To illustrate, sci-fi is distinct in its ability to alienate people from their habitual environment and engage them in the scientific, futuristic, and technological imaginaries (Ekem, 1990). Typical sci-fi prototypes involve futuristic cities, space exploration, extraterrestrial life, parallel universes, time travel, human enhancement technology, and investigating how human society might be impacted by and cope with plausibly impossible change (Heinlein, 1991). For this reason, it has been described as a popular mode of observing the technoscientific world and a thought experiment tool to provide insights and reflections for human society (Gil, 2018; Weiner et al., 2018). Sci-fi has become a recognizable and legitimate genre in pop-culture studies, not only from the critical literature perspective but also from cultural and social science perspectives (for a review, see Menadue and Cheer, 2017). In this study, we define sci-fi as a specific genre of literature and audio-visual texts that envision and portray alternative worlds or extraordinary events based on the logic of deducible scientific discoveries and technological development. Sci-fi engagement is defined as people’s daily participation with sci-fi in all media forms, including films, literature, TV series, and video games, reflecting their general consumption of, preference for, familiarity with, and mental involvement with the sci-fi genre.

Though pilots, protagonists, stories lines, and ideologies may differ across various sci-fi works, the genre takes humanity as its metacharacter and the possible human fate as its metanarrative, in contrast with any other popular genres. As literature studies have figured out, sci-fi places human conditions in the heart of discussion, and is powerful in its ability to connect us with human issues (Popper, 2022). Not only does it reflect the basic and universal needs of human beings to live and survive, but also the shared desire for all human societies to develop and thrive. For instance, sci-fi portrays how the living conditions of human species might change (e.g., *The Wandering Earth* [2019]), how humans search for the extraterrestrial homes (e.g., *Passengers* [2016]), or cope with the threats from non-human out-groups such as aliens and robots (e.g., *The Three Body Problem* [2006−2010]; *I, Robot* [2004]). It also seeks to understand how emerging technologies could enhance human bodies on the one hand, and create ethical problems on the other (e.g., *Lucy* [2014]; *Do Androids Dream of Electric Sheep?* [1968]), and how human society would develop or dysfunction in the alternative settings (e.g., *Brave New World* [1931]; *Black Mirror* [2011−2019]). Although sci-fi prototypes are imaginative, they are rooted in real-world concerns related to all human beings at present. Through analyzing the frequency of words related to disease (i.e., epidemics, pandemics, plagues, viruses, and disease) in science fiction magazines, Menadue (2020) found that representations of disease in sci-fi correlated with real-world historical trends, supporting that sci-fi appears to reflect and express contemporary human concerns and interests. Additionally, sci-fi works also convey anxiety about the emerging real-world ecological problems (e.g., climate change, technological hazard, overpopulation; Kitzinger, 2010; Otto, 2012; Rumpala, 2021), show existing social problems that violate human rights (e.g., totalitarian politics, social stratification, technological surveillance; Dongmei and Xu, 2018; Jones and Paris, 2018; Milne et al., 2021), and advocate universal humanitarian values that may help in building global solidarity (e.g., multiculturalism, cosmopolitanism; Addison-Smith, 2005; Gunderman, 2020). In this regard, sci-fi imaginaries, including both utopian and dystopian versions, might serve as a creative tool for global citizenship education by providing prospective envisioning, and proactive planning for the collective future (Starkey, 2012; Montiel et al., 2018; Doyle, 2020).

Ample studies demonstrate that narrative fictions can directly and powerfully influence people, including their identification, based on the narrative persuasion approach. This approach argues that, through being mentally transported into the fictional world, audiences may align their identification, beliefs and desires with the protagonists, experience the congruent events, thoughts and emotions, and thus, get affected by the explicit persuasive information or implicit moral conveyed by the stories (Green and Brock, 2000). Therefore, narrative fiction can yield various real-world outcomes without necessarily providing “true” information, such as evoking issue-relevant thinking (e.g., Zwarun and Hall, 2012; Hoeken and Fikkers, 2014; Borum Chattoo and Feldman, 2017), cultivating story-specific or genre consistent beliefs and worldviews (e.g., Zwarun and Hall, 2012; Xu and Kochigina, 2021), and influencing people’s attitudes, intentions and behaviors (for a review, see Braddock and Dillard, 2016). More importantly and more specified to the current study, according to the temporary expansion of the boundaries of the self (TEBOTS) model proposed by Slater et al. (2014), narrative fiction can relieve people from the constraints of their given identities, permitting them to take the perspectives of the members from a certain social group, and thus expand the boundaries of their personal and social selves (Johnson et al., 2014, 2016). Gabriel and Young (2011) also demonstrated that experiencing a narrative can directly foster a sense of belonging to the collective portrayed by the stories, facilitating the process of collective assimilation. Specifically, reading a passage from *Harry Potter and the Sorcerer’*s *Stone* led participants to feel like a part of the wizard community, and reading a passage from *Twilight* led participants to feel like a part of the vampire community (Gabriel and Young, 2011). Likewise, by engaging in sci-fi narratives, people may align their perspectives and standpoint with all of humanity, connect themselves to the human collective, vicariously experience the possible scenarios of human fate, perceive the shared needs and desires of all humanity, have concern for universal human rights involved in the imaginative or realistic global issues, endorse the human values conveyed by the narratives, and thus assimilate the human identity into self-concept, and develop their IWAH. Though previous studies have mostly examined identity formation as an immediate consequence of fictional viewing in experimental settings, accumulated fiction engagement can also cultivate long-lasting beliefs, worldviews, and social perceptions, as well as moral values (Appel, 2008; Bilandzic and Busselle, 2012; Young, 2019). In this regard, people who engage in sci-fi may not only be transported into sci-fi narratives frequently, but also see the world through the lens of sci-fi (Gierzynski, 2018; Young and Carpenter, 2018). That is, they link the concept of humanity from abstract to concrete, perceive their bond with human beings, and get cognitively and emotionally involved in issues concerning all humans. Thus, we hypothesized that:

**H1:** Sci-fi engagement is positively associated with IWAH.

### The indirect effect via abstract construal

While the narrative persuasion approach, which has been widely examined by fiction studies, suggests a direct link between sci-fi engagement and IWAH, the cognitive-literary approach may provide a more subtle and unique explanation for this association. The cognitive-literary approach bridges literature studies and cognitive science, and concerns the cognitive process in and cognitive outcomes of literature consumption (Oatley, 2012; Burke and Troscianko, 2017). Various studies have suggested that fiction consumption in general or in a specific genre may impact people’s cognitive abilities or cognitive tendencies, such as theory-of-mind and empathy, regardless of the specific content (Kidd and Castano, 2013; Mikkonen, 2015; Dodell-Feder and Tamir, 2018). In light of this and drawing upon the construal level theory (CLT), we predicted that sci-fi engagement would be associated with abstract construal, which in turn, could positively predict IWAH.

Construal level theory assumes that people tend to mentally construct an object or event from an ego-centric perspective, resulting in a continuum of construal levels from low to high abstraction (Trope and Liberman, 2003). For instance, people can construe “making a list” either concretely as “writing things down,” or abstractly as “get organized.” People leverage their abstract (vs. concrete) construal to encode and retrieve information that is temporally, spatially, socially, and hypothetically distant from (vs. close to) their direct experience (Trope and Liberman, 2010). When in an abstract (vs. concrete) mindset, people adopt a holistic (vs. narrow) view, and place greater emphasis on the general (vs. specific) aspects, long-term (vs. immediate) consequences, and superordinate (vs. subordinate) goals of the events, and thus form sequential perception, judgement, evaluation, and decision (Trope and Liberman, 2011). While levels of construal can be situationally activated, it is also viewed as a personal trait which reflects people’s cognitive inclination of abstract processing style (Vallacher and Wegner, 1989). The current study examines the mediating role of abstract construal as an individual difference.

As informed by the core assumption of the CLT, the level of abstraction increases along with psychological distance. As a genre of “cognitive estrangement,” sci-fi creates extraordinary media experiences through engaging people in alternative world settings comprising temporally (e.g., future), geographically (e.g., outer space), socially (e.g., aliens), and hypothetically (e.g., time travel) distant events or elements (Carney, 2017). People are required to leverage their abstract mindset to interpret the unfamiliar information and categorize the novel stimuli into their existing mental schema during exposure (Förster, 2009). Further, it is reasonable that accumulated sci-fi engagement will make the abstract construal of a person more active and accessible, compared to those who do not engage with sci-fi. Another line of studies suggested that sci-fi tends to cultivate a broad and flexible mind, which serves as the basis of abstract construal (Trope and Liberman, 2010). Specifically, sci-fi may extend people’s imaginative boundaries and scope of thought by incorporating advanced knowledge, future consequences, alternative worlds, and extreme events in to their mental schema. However, people with low sci-fi engagement may only take their limited experience and fixed reality as the only reference point when construing the world. Additionally, sci-fi exposure was found to enhance curiosity and creativity (Lin et al., 2013; Lin, 2014; KaradenİZ and DeuGİRmenÇAy, 2020), and be positively associated with openness to experience (Stern et al., 2019). Black et al. (2018) further demonstrated that familiarity with the sci-fi genre could positively predict the inclination to judge extraordinary events as possible, and morally deviant actions as acceptable, suggesting that sci-fi would help people to overcome their narrow imagination and rigid perspective. When people think broadly and flexibly, they will be more fluent in switching their perspectives and attention, consider various aspects of an idea or events, and engage in high-order conceptualization, which are linked to abstract construal (Pyone and Isen, 2011; Mrkva et al., 2018; Chan and Wang, 2019). Thus, we hypothesized that:

**H2:** Sci-fi engagement is positively associated with abstract construal.

When adopting an abstract mindset, people are inclined to engage in global processing and turn to the big picture of an event or object, which makes the superordinate goals more salient, and the contextual factors more inessential. Thus, abstract construal may engender a broad and high-order categorization, which also pertains to the judgment and evaluation of social targets, including the self (McCrea et al., 2012). Hence, it is possible that people with more abstract construal are more likely to categorize people all over the world as one, and themselves as members of the superordinate social group. In a similar vein, construal level can yield favorable outcomes for intergroup attitudes and relations. For instance, people with abstract (vs. concrete) construal would perceive greater similarities among people of different social categories, exhibit higher perspective-taking across groups, and are more motivated to help those in need, even if they are socially stigmatized (Levy et al., 2002). Additionally, abstract (vs. concrete) construal may serve to reduce intergroup prejudice (Luguri et al., 2012), promote moral inclusion (Mentovich et al., 2016), and is associated with preference for cooperation rather than competition in the conflict management context (Mukherjee and Upadhyay, 2019). Through broadening people’s attention to the gestalt wholes, abstract construal also promotes decisions to maximize joint outcomes of the collective unit, regardless of the beneficiary (Stillman et al., 2018). Thus, we hypothesized that:

**H3:** Abstract construal is positively associated with IWAH.

**H4:** Abstract construal mediates the positive relationship between sci-fi engagement and IWAH.

### The moderating role of actively open-minded thinking

People may sometimes find themselves incapable of transporting into the fictional worlds or unwilling to accept the deviant moral values conveyed by the fictional works (Black and Barnes, 2017). Indeed, sci-fi, as a tool of thought experiment, may carry knowledge, beliefs, and even values that inevitably contradict and challenge people’s existing mental schema, causing cognitive dissonance. In this regard, we proposed AOT as a boundary condition for the relationship between sci-fi engagement, abstract construal, and IWAH.

As a dispositional cognitive style, AOT refers to a tendency and an epistemic motivation to seek out and consider new, though potentially threatening evidence, opinions, beliefs, and values (Price et al., 2015; Kahan and Corbin, 2016). People with high AOT are motivated to collect information to prevent subconscious bias from their pre-existing standpoints (Haran et al., 2013; Mellers et al., 2015), tend to think rationally and reflectively, and will seriously consider revising their own beliefs by fairly weighing on the alternative information source (Yilmaz and Saribay, 2017; Janssen et al., 2020). On the contrary, people with low AOT tend to defend their pre-existing beliefs, and spare little effort and time in processing the evidence or opinions that contract with their owns. Empirical studies have suggested that AOT may reduce myside bias in reasoning and judgement. Specifically, compared to close-minded people, open-minded people are less influenced by their pre-existing beliefs when evaluating the quality of the arguments (Stanovich and West, 1997), exhibit less hostility towards the counterarguments against their own opinion (Stanovich and West, 2008), and respond in a more conciliative fashion to peer disagreement (Beebe and Matheson, 2021). For this reason, AOT is thought to be a qualified antidote to the epistemic or ideological rejection to complex and controversial science. For instance, Sinatra et al. (2003) found that, compared to learners with low AOT, those who score high in AOT report greater understanding of evolution theory, and an inconclusive and changing scientific belief. Stenhouse et al. (2018) also found that people with higher AOT are more likely to adopt the belief of human-caused climate change, regardless of their political ideology.

Considering the nature of AOT, it may function as an imaginative lubricant that facilitates people to adopt a more open mindset when engaging with sci-fi, a genre that typically employs hyperbole to express certain scientific beliefs or moral values in counterfactual story settings, and emphasizes the changeable facet of the world. Specifically, people with high AOT will find themselves more involved in and ponder over the fictious issues concerning the collective fate of human community (e.g., alien invasion, apocalypse, robots taking over the world), even though there is little chance that these events might become reality. In contrast, people with low AOT will treat the plausibly impossible events as an epistemic threat to their ingrained belief of the world’s being just, orderly, and stable (Lerner and Miller, 1978), and thus, may resist to transform their vicarious experiences in sci-fi into real-world concerns and intentions, inhibiting the formation of IWAH. Supporting this argument, Feinberg and Willer (2011) found that people exhibited more global-warming skepticism and less willingness to reduce carbon footprint when they were exposed to a dire message that conveys apocalyptic imaginaries of global warming. Thus, we hypothesized that:

**H5:** AOT positively moderates the effect of sci-fi engagement on IWAH, such that the direct effect of sci-fi engagement on IWAH will be stronger for people with high AOT.

Likewise, people with high AOT will actively seek out and consider for the counterfactual situations or even deviant moral values embedded in sci-fi prototypes, and be more ready and open to categorize them into their existing intellectual, cognitive, and moral structures, which may boost the effect of sci-fi engagement on a broad mind, namely, abstract construal. However, people with low AOT may consciously avoid to engage in the complex, controversial, and challenging concepts in sci-fi to reduce cognitive load and epistemic conflicts, and would only focus on the details that fall into their expectations or pre-existing beliefs. Thus, the effect of sci-fi engagement on abstract construal will be weaker for those with lower AOT.

**H6:** AOT positively moderates the effect of sci-fi engagement on abstract construal, such that the effect of sci-fi engagement on abstract construal will be stronger for people with high AOT.

**H7:** AOT positively moderates the indirect effect of sci-fi engagement on IWAH via abstract construal, such that the indirect effect of sci-fi engagement on IWAH via abstract construal will be stronger for people with high AOT.

**FIGURE 1:**
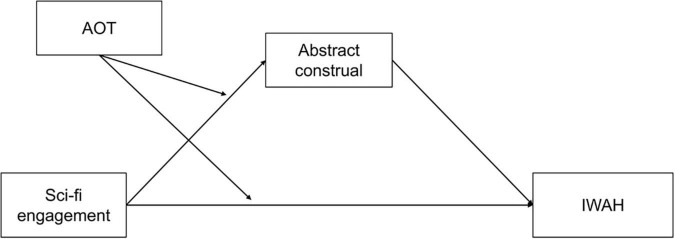
The proposed research model. IWAH, Identification with all humanity; AOT, Actively open-minded thinking.

The research model is presented in Figure 1.

## Materials and methods

### Participants

Participants were recruited using the paid sampling service provided by *Wenjuanxing*^1^, a professional online survey company in mainland China. There are over 2.9 million registered respondents with diverse demographic backgrounds in the sampling pool of *Wenjuanxing*. A considerable number of previous studies have employed this sampling strategy to investigate various social and psychological issues (e.g., Zheng and Zheng, 2014; Ning et al., 2020). The online questionnaire was distributed and collected from January 27 to February 5, 2022. The data collection protocol was approved by the academic committee of the authors’ affiliated institution. Participants provided voluntary informed consent before they start the survey. A total number of 594 participants completed the survey, while 24 out of them failed to pass the attention check, resulting in the final sample size of 570. Table 1 presents the sample characteristics.

**TABLE 1:** Sample profile (*N* = 570).

Variables	Distribution	Frequency	Percent (%)
Gender	Male	258	45.3
	Female	312	54.7
Age	18∼25	82	14.4
	26∼30	208	36.5
	31∼35	158	27.7
	36∼40	67	11.8
	41∼45	21	3.7
	>45	34	6.0
Education level	Middle school or lower	6	1.1
	High school	15	2.6
	College	62	10.9
	Bachelor’s	441	77.4
	Master’s or higher	46	8.1
Monthly income	0∼1000 RMB	14	2.5
	1001∼3000 RMB	69	12.1
	3001∼6000 RMB	127	22.3
	6001∼10000 RMB	195	34.2
	10001∼15000 RMB	116	20.4
	More than 15000 RMB	49	8.6
Overseas experiences	Yes	142	24.9
	No	428	75.1

### Measures

All scales involved in the current study were originally developed in English, and translated into Chinese following a rigorous translation-backtranslation procedure.

#### Identification with all humanity

The measurement for IWAH was adopted from the 9-item scale developed by McFarland et al. (2012). The original scale assessed people’s identification at three levels: local community, country, and humanity. Given the interest of the current study, we only kept the humanity level as previous studies did (e.g., Sparkman and Hamer, 2020). Sample items include, “How close do you feel to people all over the world,” and “When they are in need, how much do you want to help people all over the world.” Participants were asked to rate on a 5-point Likert scale from 1 (not at all) to 5 (very much). Confirmatory factor analysis (CFA) indicated that one-factor model fit with the current data satisfactorily (χ^2^= 122.342, *df* = 27, χ^2^/*df* = 4.531, CFI = 0.943, TLI = 0.923, RMSEA = 0.079,SRMR = 0.041), which is consistent with a previous study conducted in China (Deng, 2021). Thus, we averaged the 9 items to index people’s identification with all humanity, and the reliability was satisfactory (Cronbach’s α = 0.86).

#### Abstract construal

The 25-item Behavioral Identification Form (BIF; Vallacher and Wegner, 1989) was adopted to measure chronic abstract construal. Participants were asked to choose between two options to describe 25 activities either in terms of concrete (coded as 0), or abstract manner (coded as 1). For instance, participants can choose either “putting a key in the lock” (concrete construal) or “securing the house” (abstract construal) to indicate their preference to describe “locking a door.” CFA showed that the model fit was satisfactory (χ^2^= 508.927, *df* = 275, χ^2^/*df* = 1.851, CFI = 0.936, TLI = 0.930, RMSEA = 0.039, SRMR = 0.038). The reliability of the scale was assessed through Kuder–Richardson Formula 20 (KR-20), which is equivalent to Cronbach’s alpha for dichotomously scored items (Kuder and Richardson, 1937). The KR-20 coefficient of 0.90 indicated a good reliability. Responses for each item were summed up to form the total BIF score, with higher score indicating more abstract construal.

#### Sci-fi engagement

Sci-fi engagement was measured through the 12-item Science Fiction Hobbyism Scale developed by Koverola et al. (2020), reflecting people’s consumption of, preference for, familiarity with, and mental involvement in sci-fi genre in general. Participants were first provided with the following brief definition of sci-fi for the current study: “Science fiction refers to a specific genre of literature, movies, televisions, games, and comics that portrays alternative worlds or extraordinary events based on the logic of deducible scientific discoveries and technological development. Typical themes or elements of sci-fi genre may include space exploration, futuristic technology, time and space travel, posthuman (e.g., superman, robots, aliens, human cloning, cyborg), and ecological crises (e.g., virus, apocalypse, energy shortage).” Next, they were asked to complete the items on a 7-point Likert scale from 1 (strongly disagree) to 7 (strongly agree). Sample items were: “I have spent a lot of time on sci-fi movies, literature, games, TV shows and/or comics,” and “I often think about what machines are going to be like in the future.” Though the reliability of the original scale was good (Cronbach’s α = 0.90), CFA indicated that the scale did not fit our data satisfactorily (χ^2^= 694.858, *df* = 54, χ^2^/*df* = 12.867, CFI = 0.806, TLI = 0.762, RMSEA = 0.144, SRMR = 0.074). Thus, we considered omitting some items to improve the construct validity. Following the guide of previous studies (e.g., Voss et al., 2003; Fish et al., 2010), we iterated the process of deleting one item at one time based on factor loadings or recommendation of modification indices (MIs) and re-estimating the measurement model until the model fit was acceptable. One item (i.e., “For me, science fiction is an interesting topic”) was firstly excluded due to low factor loading (<0.5), and three items (i.e., “I have actively participated in conventions and gatherings related to science fiction,” “I try to keep up to date on technological and scientific advances,” and “I often think about things related to artificial intelligence”) were then dropped based on the recommendation of MIs, which suggested the possibility of item redundancy. The removal of the four items resulted in an acceptable model fit (χ^2^= 125.069, *df* = 20, χ^2^/*df* = 6.253, CFI = 0.936, TLI = 0.910, RMSEA = 0.096, SRMR = 0.042). Finally, the retained 8 items were averaged to index sci-fi engagement, and the reliability was good (Cronbach’s α = 0.86).

#### Actively open-minded thinking

We adopted the 7-item scale used by Haran et al. (2013); Mellers et al. (2015) to assess AOT. Sample items are: “People should take into consideration evidence that goes against their beliefs,” and “One should disregard evidence that conflicts with one’s established beliefs (reverse coded).” Participants responded on a scale from 1 (completely disagree) to 7 (completely agree). Though the reliability of this measure for the current sample is acceptable (Cronbach’s α = 0.61) and comparable to previous studies such as Kahan and Corbin (2016); Svedholm-Häkkinen and Lindeman (2018), both of which report α = 0.61 for this measure, CFA indicated that the initial scale did not fit well with our data (χ^2^= 136.237, *df* = 14, χ^2^/*df* = 9.731, CFI = 0.775, TLI = 0.663, RMSEA = 0.124, SRMR = 0.077). However, the removal of two items with factor loadings below 0.5 (i.e., “People should take into consideration evidence that goes against their beliefs,” “People should revise their beliefs in response to new information or evidence”) could simultaneously increase model fit (χ^2^= 16.895, *df* = 5, χ^2^/*df* = 3.379, CFI = 0.972, TLI = 0.945, RMSEA = 0.065, SRMR = 0.025) and reliability (Cronbach’s α = 0.66). Thus, the retained five items were averaged to index AOT after reverse coding.

#### Control variables

Gender was assessed as a dichotomous variable (female = 0, male = 1; 45.3% males) and age as a continuous variable (*M* = 31.86, *SD* = 7.41). Education level (middle school or lower = 1, high school = 2, college = 3, bachelor’s = 4, master’s or higher = 5; Median = 4, or Bachelor’s degree) and monthly income (0∼1000 RMB = 1, 1001∼3000 RMB = 2, 3001∼6000 RMB = 3, 6001∼10000 RMB = 4, 10001∼15000 RMB = 5, More than 15000 RMB = 6; Median = 4, or 6,001∼10,000 RMB) were both assessed as ordinal variables. Additionally, considering the international personal contact would impact IWAH (Römpke et al., 2019), we also controlled for overseas experiences by asking participants to indicate whether they have had experiences of living, studying, or working abroad (0 = No, 1 = Yes; 24.9% Yes).

#### Statistical analyses

To examine common method bias, an exploratory factor analysis (EFA) was conducted using SPSS 25.0, and a series of CFAs were conducted using Mplus 8.3. Then, we used SPSS 25.0 to calculate the means, and standard deviations of the variables, and examined their inter-correlations. After all the variables were mean-centered, we leveraged the PROCESS version 3.5 to test the research hypotheses. The PROCESS Model 4 was employed to assess the mediating role of abstract construal, while the Model 8 was employed to assess the moderating role of AOT in the direct and indirect paths. The mediation effect and moderated mediation effects were tested with 5,000 bootstrap samples (Hayes and Scharkow, 2013), and were established if the 95% bias-corrected confidence interval did not include zero.

## Results

### Preliminary analysis

To avoid the common method bias caused by the self-report questionnaire, participants were informed of the confidentiality principle and answered the questions anonymously, including the reversed items. Additionally, we conducted a series of statistical analyses to test the common method bias. First, we relied on Harman’s one-factor method, which assumes that a common method bias is present if only one factor was extracted, or a single factor explains over 50% of the total variance in the factor analysis. (Podsakoff and Organ, 1986; Podsakoff et al., 2003). We conducted an un-rotated EFA with all scale items entered. The EFA extracted 9 factors with an eigenvalue greater than one, and the first factor explained 19.78% of the overall variance, less than the critical value of 50%, indicating the common method was acceptable.

We conducted a series of CFAs to further rule out the common method bias. Model fit indices demonstrated that the four-factor measurement model (i.e., 9-item IWAH, 25-item abstract construal, 8-item sci-fi engagement, and 5-item AOT) yielded an acceptable model fit, while the alternative models, including the one-factor model, yielded poor model fits (see Table 2). Therefore, the common method was not a problem in this study (Podsakoff et al., 2012).

**TABLE 2:** Measurement model comparison.

	*χ^2^*	*df*	*χ^2^/df*	CFI	TLI	RMSEA	SRMR
Four-factor model	1761.671	1028	1.714	0.906	0.901	0.035	0.048
Three-factor model 1^a^	2135.990	1031	2.072	0.859	0.852	0.043	0.053
Three-factor model 2^b^	2203.529	1031	2.137	0.850	0.843	0.045	0.058
Three-factor model 3^c^	3247.613	1031	3.150	0.717	0.703	0.061	0.088
Two-factor model ^d^	3680.763	1033	3.563	0.662	0.646	0.067	0.093
One-factor model ^e^	5037.302	1034	4.872	0.489	0.466	0.082	0.108

*N* = 570. χ^2^, chi-square discrepancy; df, degrees of freedom; CFI, comparative fit index; TLI, Tucker–Lewis index; RMSEA, root mean square error of approximation; SRMR, standardized root mean square residual.

^*a*^Actively open-minded thinking and sci-fi engagement were combined into one factor.

^*b*^Abstract construal and actively open-minded thinking were combined into one factor.

^*c*^Abstract construal and sci-fi engagement were combined into one factor.

^*d*^Abstract construal, actively open-minded thinking, and sci-fi engagement were combined into one factor.

^*e*^Abstract construal, actively open-minded thinking, sci-fi engagement, and identification with all humanity were combined into one factor.

Table 3 displays the means, and standard deviations of the variables, and their inter-correlations. The results showed that sci-fi engagement positively correlated with IWAH, and abstract construal. Sci-fi engagement positively correlated with abstract construal. Contrarily, AOT negatively correlated with IWAH and sci-fi engagement.

**TABLE 3:** Descriptive statistics and correlations of variables.

Variable	*M*	*SD*	1	2	3	4
(1) IWAH	3.37	0.69	–			
(2) Abstract construal	16.18	6.30	0.21**	–		
(3) Sci-fi engagement	4.86	1.04	0.45**	0.23**	–	
(4) AOT	4.82	0.98	−0.12**	−0.04	−0.13**	–

**p < 0.01; IWAH, Identification with all humanity; AOT, Actively open-minded thinking; M, Mean; SD, Standard deviation.

### Testing the mediation model

**FIGURE 2:**
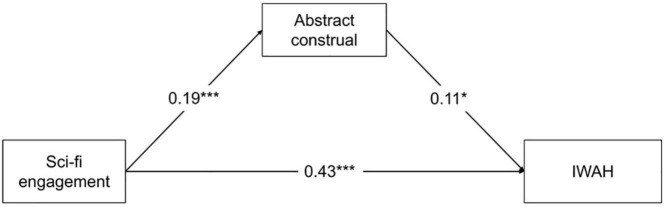
Results of the mediation analysis. ***p < 0.001, *p < 0.05; IWAH, Identification with all humanity.

We employed PROCESS Model 4 to assess the mediating role of abstract construal between sci-fi engagement and IWAH. Gender, age, education level, monthly income and overseas experience were entered as the covariates in the analyses. Table 4 presents the results of regression tests. The results indicated that sci-fi engagement was positively associated with IWAH (β = 0.45, SE = 0.04, *t* = 11.27, *p* < 0.001). Thus, H1 was supported. Consistent with H2 and H3, sci-fi engagement was positively associated with abstract construal (β = 0.19, SE = 0.04, *t* = 4.33, *p* < 0.001), and abstract construal was positively associated with IWAH (β = 0.11, SE = 0.04, *t* = 2.86, *p* < 0.01). More importantly, abstract construal positively mediated the effect of sci-fi engagement on IWAH (β = 0.02, SE = 0.01, 95%CI [0.007, 0.043]), supporting H4. Additionally, after abstract construal was incorporated as mediating variable into the model, the positive predictive effect of sci-fi engagement on IWAH was still significant (β = 0.43, SE = 0.04, *t* = 10.64, *p* < 0.001). Thus, abstract construal partially mediated the positive effect of sci-fi engagement on IWAH, and the mediation effect accounted for 4.44% (0.02/0.45) of the total effect. Figure 2 displays the results of the mediation analysis.

**TABLE 4:** Testing the mediation effect of abstract construal between sci-fi engagement and identification with all humanity (IWAH).

Predictors	Model 1 (IWAH)		Model 2 (Abstract construal)		Model 3 (IWAH)	
	β	*t*	β	*t*	β	*t*
Gender	−0.09	−2.44*	0.04	0.92	−0.10	−2.57*
Age	−0.01	−0.13	0.02	0.52	−0.01	−0.20
Education	−0.06	−1.56	−0.05	−1.06	−0.06	−1.44
Income	0.01	0.16	0.11	2.33*	−0.01	−0.12
Overseas experiences	0.09	2.22*	0.03	0.59	0.08	2.17*
Sci-fi engagement	0.45	11.27***	0.19	4.33***	0.43	10.64***
Abstract construal					0.11	2.86**
R^2^	0.22		0.07		0.23	
F	26.19***		6.93***		23.91***	

***p < 0.001; **p < 0.01; *p < 0.05. IWAH, Identification with all humanity.

### The moderating role of actively open-minded thinking

**FIGURE 3:**
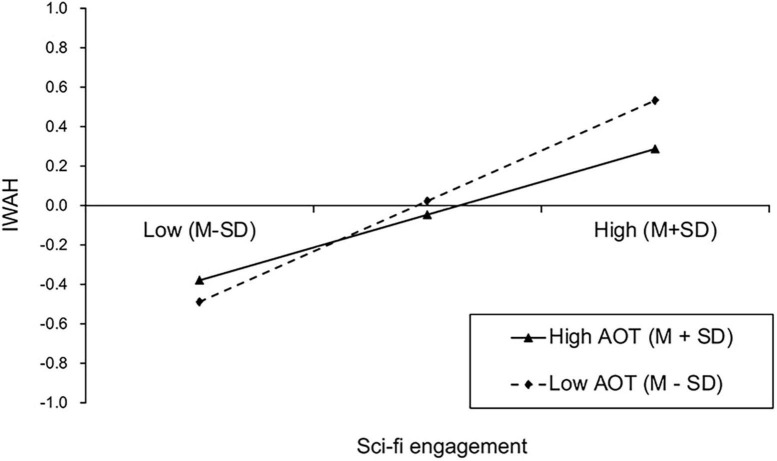
Interaction effect of sci-fi engagement and AOT on IWAH. IWAH, Identification with all humanity; AOT, Actively open-minded thinking; M, Mean; SD, Standard deviation.

**FIGURE 4:**
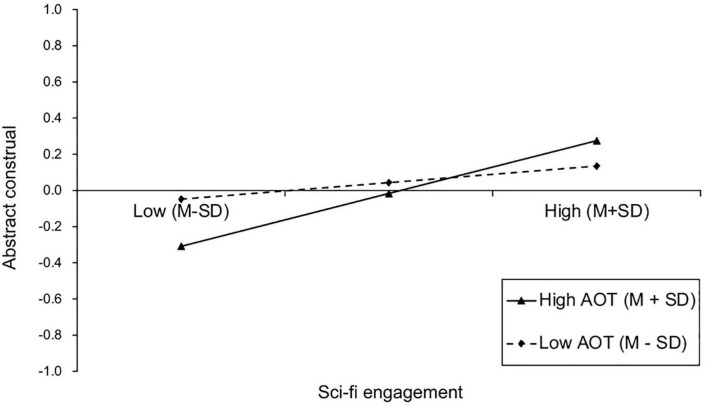
Interaction effect of sci-fi engagement and AOT on abstract construal. AOT, Actively open-minded thinking; M, Mean; SD, Standard deviation.

**FIGURE 5:**
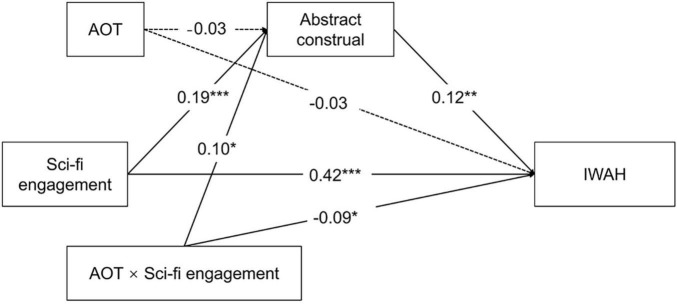
Results of the moderated mediation analysis. ***p < 0.001, **p < 0.01, *p < 0.05; IWAH, Identification with all humanity; AOT, Actively open-minded thinking.

The PROCESS Model 8 was used to test the moderating role of AOT in the direct association and indirect association via abstract construal between sci-fi engagement and IWAH. Gender, age, education level, monthly income and overseas experience were also entered as the covariates. Table 5 displays the results of the regression tests. It was found that the interaction of sci-fi engagement with AOT negatively predicted IWAH (β = −0.09, SE = 0.04, *t* = −2.37, *p* < 0.05), but positively predicted abstract construal (β = 0.10, SE = 0.04, *t* = 2.44, *p* < 0.05). The interaction effect of sci-fi engagement with AOT on IWAH was plotted in Figure 3. The results of simple slope analysis revealed that, the effect of sci-fi engagement on IWAH was stronger for people with low AOT (β_*low–AOT*_ = 0.51, SE = 0.05, *t* = 9.38, *p* < 0.001), compared to those with high AOT (β_*high–AOT*_ = 0.33, SE = 0.06, *t* = 5.90, *p* < 0.001). Thus, H5 was not supported. The interaction effect of sci-fi engagement with AOT on abstract construal was plotted in Figure 4. The results of simple slope analysis revealed that, the positive effect of sci-fi engagement on abstract construal was significant for people with high AOT (β_*high–AOT*_ = 0.29, SE = 0.06, *t* = 4.80, *p* < 0.001), but was nonsignificant for people with low AOT (β_*low–AOT*_ = 0.09, SE = 0.06, *t* = 1.53, *p* = 0.126). Thus, H6 was supported. Furthermore, the indirect effect of sci-fi engagement on IWAH via abstract construal was conditional on AOT (β = 0.01, SE = 0.01, 95%CI [0.002, 0.029]). Specifically, the indirect effect via abstract construal was significant for people with high AOT (β_*high–AOT*_ = 0.03, SE = 0.01, 95%CI [0.012, 0.071]), but was nonsignificant for people with low AOT (β_*low–AOT*_ = 0.01, SE = 0.01, 95%CI [−0.006, 0.032]). Thus, H7 was supported. Figure 5 displays the results of the moderated mediation analysis.

**TABLE 5:** Testing the moderated mediation effect of actively open-minded thinking (AOT).

Predictors	Model 1 (Abstract construal)		Model 2 (IWAH)	
	β	*t*	β	*t*
Gender	0.04	0.84	−0.09	−2.49*
Age	0.01	0.34	−0.01	−0.18
Education	−0.05	−1.07	−0.05	−1.39
Income	0.10	2.15*	0.01	0.12
Overseas experiences	0.02	0.51	0.08	2.08*
Sci-fi engagement	0.19	4.34***	0.42	10.33***
AOT	−0.03	−0.71	−0.03	−0.88
Sci-fi engagement × AOT	0.10	2.44*	−0.09	−2.37*
Abstract construal			0.12	3.10**
R^2^	0.08		0.24	
F	5.98***		19.67***	

***p < 0.001, **p < 0.01, *p < 0.05. IWAH, Identification with all humanity; AOT, Actively open-minded thinking.

## Discussion

This study proposed a moderated mediation model to assess to the effect of sci-fi engagement on IWAH, as well as the mediating role of abstract construal and the moderating role of AOT. Supporting our hypotheses, higher sci-fi engagement is associated with increased IWAH both directly, and indirectly via abstract construal. Further, AOT can facilitate the positive effect of sci-fi engagement on abstract construal, as well as the indirect relationship between sci-fi engagement on IWAH via abstract construal. However, inconsistent with our hypothesis, the positive direct effect of sci-fi engagement on IWAH was attenuated, rather than strengthened, with the increase of AOT. Two points of the results need further discussions.

Firstly, the mediation effect of abstract construal only accounted for approximately 4.44% of the total effect, which may exhibit somewhat low explanatory power and need to be cautiously interpreted. However, we argue that this indirect effect is worthy of considering, since not all sci-fi works directly portray or explicitly convey concerns for human community (e.g., *The Time Traveler’s Wife* [2009]), and abstract construal may help to explain a more stable and universal mechanism across all sci-fi works, since all sci-fi tends to alienate people from reality. Additionally, the indirect effect via abstract construal might be less susceptible to political orientation, national identity, or cultural value orientation, since it shapes people’s way of construing the world through dealing with the cognitive level, which may cause less direct psychological reactance and ideological rejection to the persuasive attempts of sci-fi. Further, we identified a boundary condition that may boost the effect of sci-fi engagement on IWAH via abstract construal: AOT. That is, this mediation effect may be enlarged for people who tend to consider for and elaborate counter-belief events and values, or when people are more epistemically open-minded in their sci-fi engagement.

Secondly, AOT intriguingly attenuated, rather than magnified, the direct association between sci-fi engagement and IWAH, which suggests the opposite direction against our hypothesis. We explained this unexpected and counterintuitive result by revisiting the nature of AOT, and its possible alternative effects on people’s mindset in sci-fi engagement. Theoretically, AOT signals the individual difference in the openness to the counter-belief values and opinions, while human identity, as an inclusive identity, salient by the sci-fi narratives does not violate people’s existing identities, and most people would endorse, rather than oppose to, the humanitarian values. In other words, sci-fi may increase people’s IWAH by highlighting their existing identification, and amplifying their inherent concern for human, rather than persuading them to adjust their values and beliefs towards ones that contradict with their owns. This would explain why the positive moderating effect of AOT in the direct association was not observed. Further, the core of AOT is rational and reflective thinking (Baron, 2019; Briki, 2022). It actually signals *a willingness to consider* a variety of intellectual perspectives (Price et al., 2015, p. 1488), rather than a tendency to be directly impacted or persuaded by whatever they see and hear (Stenhouse et al., 2018). People with high AOT tend to evaluate the merits of the information, while people with low AOT are likely to use their heuristics (i.e., mental shortcuts) in information processing (Campitelli and Gerrans, 2014). Thus, compared to people with high AOT, people with low AOT (those who score high in items like “intuition is the best guide in making decisions”) would be more responsive to the emotional appeal (e.g., fear, hope, compassion) in sci-fi narratives that attempt to raise people’s concern for humanity, and rely more on their direct media experiences, rather than rational evaluation of the possibilities of the counterfactual events, in their judgment and decision making. This would help explain why the direct persuasive effect of sci-fi would be stronger for people with low AOT. Additionally, AOT might also be associated with selective attention to the sci-fi. Previous studies found evidence that people with high AOT also score high in need for cognition, namely, the interest and enjoyment in dealing with the abstract, and complex ideas (Haran et al., 2013). Thus, compared to people with low AOT, people with high AOT (i.e., those who enjoy in thinking) would be more motivated by their epistemic needs, and might allocate more cognitive resources to seek for and elaborate intellectual elements such and scientific knowledge and technological logics bolstering the sci-fi narratives, rather than the non-intellectual elements such as the plots, characters, or the affective appeal of the sci-fi stories. Thus, it might be not that surprising that AOT, an epistemic motivation, may discount the direct persuasiveness of sci-fi, while exaggerating the indirect effect via abstract construal. The paradoxical role of AOT in moderating the direct and indirect interestingly challenges our common wisdom, which might be informative and inspiring to observe how people negotiate their complex media experiences in sci-fi engagement. However, more researches are needed to further illustrate and reconcile the paradoxical role of AOT.

### Theoretical implications

First and foremost, this study contributes to the IWAH literature by revealing its possible pop-cultural and cognitive origins. During the period of this study, the human society seem to be more turbulent than the past decades. Global crises such as COVID-19, and the Russia-Ukraine conflicts once again called on our attention to human solidarity and mutual care across subordinate social groups. While it is important to investigate the social implications of IWAH, it is also critical to know how individuals’ IWAH could be cultivated or enlarged, which has received surprisingly less attention (Loy and Reese, 2019; McFarland et al., 2019). To this end, we theorized and examined sci-fi engagement and abstract construal as antecedents of IWAH and provided empirical evidence. The findings implied that individuals’ IWAH may vary from their daily pop-cultural engagement and cognitive style in addition to previously identified variables such as personality and moral values (McFarland et al., 2012; Hamer et al., 2019), suggesting new a direction for cultivating IWAH. Of note, we should caution drawing causal conclusion of this trend, since the cross-sectional evidence only gives a first hint. It is also possible that people with high IWAH tend to adopt an abstract mindset, and are inclined to engage in sci-fi frequently. To shed light on this possibility, we conducted a series of post-hoc analyses with IWAH as the independent variable and sci-fi engagement as the dependent variable. Though supplementary analyses suggested the possibility of the reverse relationships, the predictive effect of IWAH on sci-fi engagement was weaker than that of sci-fi engagement on IWAH, and the indirect effect of abstract construal was slightly weaker in the reverse model (IWAH → abstract construal → sci-fi engagement) compared to that in the original model (sci-fi engagement → abstract construal → IWAH) when controlling for age, gender, education level, monthly income, and overseas experiences (see Supplementary Materials). Future studies may investigate the reciprocal relationships between the variables.

Secondly, this study contributes to the meaningful entertainment studies, and highlights the uniqueness of sci-fi genre. Apart from hedonic values, there is a trend suggesting that popular media content can facilitate self-transcendence and yield pro-social outcomes (Janicke and Oliver, 2017; Oliver et al., 2018; Clayton et al., 2021). Echoing this trend, this study particularly investigated the potential social implications of sci-fi, a genre that has gained worldwide popularity, and provide preliminary evidence on how it correlates with people’s tendency to transcend their subordinate social identities, thereby highlighting the pro-social aspects of entertainment narratives (Papa and Singhal, 2009; Frank and Falzone, 2021). Beyond previous researches, we conceptualized sci-fi as a gerne of humanity, and extended the scope of narrative persuasion literature from the effect of specific storytelling to the metanarrative of a genre in general, and from immediate effect to accumulated effect. Consistent with Appel (2008),we contributed to the evidence that the metanarrative of a genre is associated with the cultivation of genre-consistent meta-beliefs and meta-worldviews. Furthermore, we contribute to the conceptualization of sci-fi as “a genre of cognitive estrangement” (Suvin, 1972) by revealing its association with abstract construal, a cognitive tendency which lead people to adopt self-distancing perspectives and focus on the big picture. Indeed, Carney (2017) has also noticed the potential of sci-fi in eliciting abstract construal, and put forward the propositions that readers may exhibit high-order expectation (i.e., purposiveness, moral engagement, formal speech) related to abstract construal for sci-fi works. However, he treated abstract construal as an in-text mindset during exposure, and did not test his propositions empirically. Extending his propositions, our results indicated that abstract construal may also be viewed as a trait-like cognitive style that correlates with accumulated sci-fi engagement. If accumulated sci-fi engagement can lead to chronic abstract construal, we may expect a broader range of the real-world implications of sci-fi, since abstract construal is also related to various favorable outcomes such as self-control, perspective taking, and green consumption (Fujita and Carnevale, 2012; Reczek et al., 2018; Holt et al., 2021). Thus, the indirect mechanism via abstract construal may also extend the research scope of meaningful entertainment from the cognitive perspective.

Thirdly, we revealed the complex psychological mechanisms underlying sci-fi engagement. We found a direct association between sci-fi engagement and IWAH, building on the theoretical tenets of narrative persuasion, which emphasizes the emotional, affective, and experiential aspects of fiction narrative. An indirect association via abstract construal was also supported, building on the cognitive-literary approach and CLT, which emphasizes the cognitive, intellectual, and epistemic aspects of fiction narrative. Thus, it might be not that surprising that AOT, a cognitive style and an epistemic motivation that encourages people to rely less on their direct experience and more on information seeking and broad consideration, may inhibit the former approach but facilitate the latter approach. This unexpected finding might imply the dual nature of sci-fi genre as both an experiential and epistemic resource for people to make sense of the world and adjust their relationships with the imagined others, which is unique in sci-fi narratives. In this regard, we also suggest that narrative fiction may also function to revise schema, expand worldview, and alter cognitive style, in addition to its function of creating immediate emotional experiences, and satisfying psychological and emotional needs for autonomy, competence, and connectedness as acknowledged by previous studies (Tan, 2008; Slater et al., 2014; Silver and Slater, 2019).

### Practical implications

The association between sci-fi engagement and IWAH suggested that people who frequently engage in sci-fi will also hold stronger IWAH. Thus, sci-fi fandom should be regarded as a targeted group for the mobilization of global-oriented actions, since they may exhibit more global responsibility and less reactance. For instance, international non-government organizations (e.g., International Committee of the Red Cross) may consider mobilizing the sci-fi community to devote time, money, or effort to global issues (e.g., global poverty); policy propagandists may consider mobilizing them to help disseminate global-oriented policies (e.g., pro-environmental policies, international aid policies, anti-war policies). Considering the potential of sci-fi engagement in cultivating IWAH, we may also expect sci-fi to be a less costly, but widespread and efficient method for global citizenship education. If the causal effect is demonstrated by future researches, cultural policy makers, schools of all levels, media stakeholders, and online commercial platforms should work together to encourage people to consume or create sci-fi works in all media forms, especially during the period when we particularly need to unite people all over the world.

The mediating role of abstract construal in the relationship between sci-fi engagement and IWAH also provides implications for sci-fi creation and global-oriented persuasion. Specifically, writers, film makers, cartoonist, and game designers of sci-fi may consider setting their stories in the distant future, portraying scientific and technological elements that will largely broad the mind of people, and continuously exploring prototypes that will create novel media experiences, such that the effect of sci-fi on IWAH via abstract construal will be maximized. Additionally, considering the positive association between abstract construal and IWAH, we recommend global-oriented persuasion (e.g., pro-environment persuasion, international donation persuasion) emphasizing the superordinate goal, long-term consequences, and desirability of the actions, which might help to elicit abstract mindset and make people feel connected to people beyond their close relationships.

The paradoxically moderating role of AOT may imply that the match between sci-fi style and personal cognitive style matters for raising IWAH. While some sci-fi works focus on the story lines and the affective aspect, which is known as soft sci-fi, others may weigh more on the scientific and technological elements, which is known as hard sci-fi. For people with high AOT (e.g., liberals), hard sci-fi might be more effective in raising their IWAH, whereas for people with low AOT (e.g., conservatives), soft sci-fi portraying human issues might be more effective to raise their IWAH (Price et al., 2015).

### Limitations and future directions

First, the main limitation of this study is its correlational nature, which inhibited us from interpreting the causal relationship between sci-fi engagement, abstract construal, and IWAH, since the proposed relationship might be adverse or bidirectional, or an unconsidered variable caused all of them. Thus, future study may explore the effect of sci-fi engagement in experimental settings to establish the causal effect. For example, a line of studies has employed experimental design the test the effect of sci-fi exposure on acceptance of new technology, privacy concern, and political attitudes (Appel et al., 2016; Jones and Paris, 2018; Milne et al., 2021). It is also interesting to see whether exposure to sci-fi that portrays all humanity or simply displaying futuristic technology or outer space will elicit IWAH. To ascertain the temporal effect, future studies may employ follow-up design to determine the directionality of the proposed variables over time.

Second, this study was conducted in China with a non-randomized adult sample. Indeed, previous studies have found that, compared to people from western countries, Chinese people are more collectivistic, tend to adopt a holistic processing style, and exhibit higher interdependent self-construal (Hui et al., 1991; Nisbett et al., 2001; Li et al., 2006). Thus, the positive direct effect may be partially due to their way of construing the world and their selective attention to the collective narratives and community values in sci-fi narratives. To address this, future studies are encouraged to replicate the proposed model in other countries, especially in western cultures, and investigate that whether people all over the world interpret sci-fi in a shared manner. Additionally, the results should be cautiously generalized to other social groups, such as adolescents, who are excluded from the current study. Furthermore, random national sampling technique may be employed to strengthen the representativeness of the current sample.

Third, though we have identified abstract construal as a possible mediator, the proportion explained by it is relatively low. One possible reason is that the direct effect of sci-fi engagement on identity formation is so strong that it overwhelmed the indirect effect through the cognitive mechanism. Thus, future studies may employ experimental design to see whether the cognitive approach may be more salient for sci-fi narratives that do not explicitly convey the concern for all humanity, and instead purely display alternative world setting (e.g., outer space), and futuristic technologies. Also, more researches must explore alternative mediators between sci-fi engagement and IWAH from the emotional, social, and cognitive perspectives. For instance, scholars may consider global consciousness, perceived intergroup similarity, and cognitive complexity as mediators of sci-fi engagement and IWAH (Levy et al., 2002; Reysen and Katzarska-Miller, 2013; Hamer et al., 2019).

Last, the current study did not differentiate the effect of different subcategories and subthemes of sci-fi genre. Indeed, sci-fi pieces produced by different countries and social groups carry disparate visions of future worlds and aim to convey diverse values or ideologies. Some sci-fi narratives may exhibit optimistic future visions by portraying technological advancement, and human flourish, known as the utopian sci-fi, whereas others may convey strong pessimism by highlighting the disasters, totalitarian politics, as well as social stratification, known as the dystopian sci-fi. Future studies may investigate whether sci-fi narratives with different themes, emotion valence, and story structures may have different impacts on IWAH.

## Conclusion

The present study proposed a moderated mediation model to test the effect of sci-fi engagement on IWAH, as well as the mediating role of abstract construal, and the moderating role of AOT. A direct association between sci-fi engagement and IWAH was revealed, and an indirect effect of sci-fi engagement and IWAH via abstract construal was also confirmed. The direct and indirect paths were both moderated by AOT. Specifically, for people with higher AOT, the direct effect of sci-fi engagement on IWAH was weaker, whereas the indirect path via abstract construal was stronger. This is the among the first studies addressing the relationship, mechanism, and boundary condition between sci-fi engagement and IWAH, which indicates the potential of sci-fi, as a worldwide pop-culture genre, in cultivating IWAH.

## Data availability statement

The raw data supporting the conclusions of this article will be made available by the authors, without undue reservation.

## Ethics statement

The studies involving human participants were reviewed and approved by the Institutional Review Board of the Academic Committee of School of Journalism and Communication, Tsinghua University. The patients/participants provided their written informed consent to participate in this study.

## Author contributions

FW conceived the idea, carried out the study design, collected and analyzed the data, and wrote the manuscript. MZ participated in conceptualization, results interpretation, and manuscript revision. ZZ acquired the funding, supervised the whole project, and participated in conceptualization, results interpretation, and manuscript revision. All authors contributed to the article and approved the final version.

## Conflict of interest

The authors declare that the research was conducted in the absence of any commercial or financial relationships that could be construed as a potential conflict of interest.

## Publisher’s note

All claims expressed in this article are solely those of the authors and do not necessarily represent those of their affiliated organizations, or those of the publisher, the editors and the reviewers. Any product that may be evaluated in this article, or claim that may be made by its manufacturer, is not guaranteed or endorsed by the publisher.

## Footnotes

1
www.sojump.com
