# Supplementary material for: Can science fiction engagement predict identification with all humanity? Testing a moderated mediation model
Source: Front Psychol. 2022 Aug 18;13:943069. doi: 10.3389/fpsyg.2022.943069 (PMC9435529; doi:10.3389/fpsyg.2022.943069)
Supplement: Supplementary file 1 [file Data_Sheet_1.PDF]

Supplementary Materials

Post hoc analyses on the reverse relationships

Since we collected all the variables at one time, we are not able to determine the causal relationships between the variables. It is also possible that identification with all humanity (IWAH) would serve as the antecedent of sci-fi engagement, and this association could also be mediated by abstract construal.

To shed more light on these possibilities, we firstly conducted a regression analysis with IWAH as the independent variable and sci-fi engagement as the dependent variable (see Table S1). Results indicated that after controlling for gender, age, education level, monthly income, and overseas experiences, the effect of IWAH on sci-fi engagement was also significant (β=0.405, SE=0.04, p<0.001), but was weaker than that of sci-fi engagement on IWAH (β=0.454, SE=0.04, p<0.001). Following the guide of previous studies (e.g., Wei et al., 2016; Fürstenberg et al., 2021), we also conducted a post-hoc mediation analysis using PROCESS Model4 to test the reserve indirect effect (i.e., IWAH → abstract construal → sci-fi engagement) and compare it to the original indirect effect (i.e., IWAH → abstract construal → sci-fi engagement). Figure S1 displays the results of the reverse mediation model. Results indicated that when controlling for after controlling for gender, age, education level, monthly income, and overseas experiences, IWAH could also positively predict abstract construal (β=0.183, SE=0.04, p<0.001), but this effect was slightly weaker than that of sci-fi engagement on abstract construal (β=0.191, SE=0.04, p<0.001). Moreover, abstract construal could also mediate the relationship between IWAH and sci-fi engagement (β=0.018, 95%CI [0.005, 0.039]), but this reverse indirect effect was also slightly weaker than the original indirect effect (β=0.021, 95%CI [0.074, 0.044]). To conclude, though the post-hoc analyses did not rule out the possibility of the reverse relationships, they do not reject the focal conclusions drawn from the main study.

References

Wei, M., Li, C. I., Wang, C., & Ko, S. Y. (2016). Finding benefits from acculturative stress among Asian Americans: Self-reflection moderating the mediating effects of ethnocultural empathy on positive outcomes. *J. Couns. Psychol*. 63, 633-644. Doi: 10.1037/cou0000173

Fürstenberg, N., Alfes, K., & Shantz, A. (2021). Meaningfulness of work and supervisory-rated job performance: A moderated-mediation model. *Hum Resour Manage*. 60, 903-919. Doi: 10.1002/hrm.22041

Table S1. The effect on IWAH on sci-fi engagement

	**Sci-fi engagement**	
**Predictors**	β	t
Gender	0.161	4.485***
Age	-0.001	-0.025
Education	0.104	2.780**
Income	0.201	5.019***
Overseas experiences	0.065	1.747
IWAH	0.405	11.265***
R^2	0.302	
F	40.645***	

***p<0.001; **p<0.01. IWAH, Identification with all humanity.

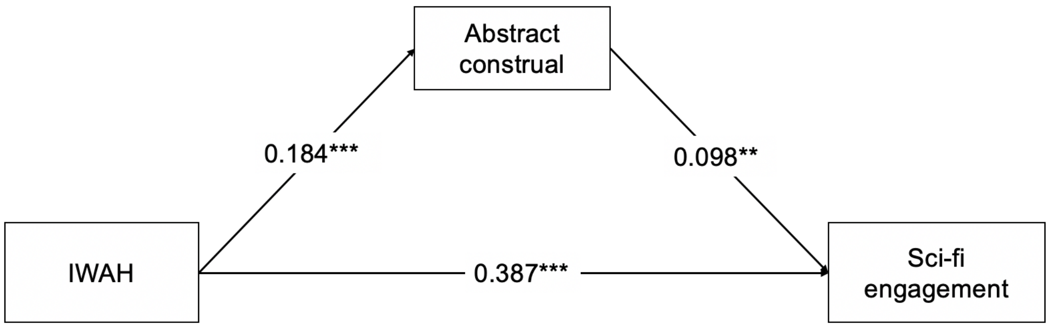

Figure S1. The effect of IWAH on sci-fi engagement via abstract construal. IWAH, Identification with all humanity.
